# Preclinical Activity of Sacituzumab Govitecan, an Antibody-Drug Conjugate Targeting Trophoblast Cell-Surface Antigen 2 (Trop-2) Linked to the Active Metabolite of Irinotecan (SN-38), in Ovarian Cancer

**DOI:** 10.3389/fonc.2020.00118

**Published:** 2020-02-12

**Authors:** Emanuele Perrone, Salvatore Lopez, Burak Zeybek, Stefania Bellone, Elena Bonazzoli, Silvia Pelligra, Luca Zammataro, Aranzazu Manzano, Paola Manara, Anna Bianchi, Natalia Buza, Joan Tymon-Rosario, Gary Altwerger, Chanhee Han, Gulden Menderes, Elena Ratner, Dan-Arin Silasi, Masoud Azodi, Pei Hui, Peter E. Schwartz, Giovanni Scambia, Alessandro D. Santin

**Affiliations:** ^1^Department of Obstetrics, Gynecology, and Reproductive Sciences, Yale University School of Medicine, New Haven, CT, United States; ^2^Department of Woman and Child Health Sciences, Universita' Cattolica del Sacro Cuore, Rome, Italy; ^3^Department of Experimental and Clinical Medicine, Magna Graecia University, Catanzaro, Italy; ^4^Department of Pathology, Yale University School of Medicine, New Haven, CT, United States

**Keywords:** sacituzumab govitecan, ovarian carcinoma, antibody drug conjugate (ADC), Trop-2, target therapy

## Abstract

**Background:** Epithelial ovarian cancer (EOC) is the most lethal gynecologic malignancy. Sacituzumab govitecan (SG) is a novel antibody-drug-conjugate (ADC) targeting trophoblast-antigen-2 (Trop-2), a cell surface glycoprotein highly expressed in many epithelial tumors, to deliver SN-38, the active metabolite of irinotecan. This study aimed to evaluate Trop-2 expression in EOC tissues and the preclinical activity of SG against primary EOC cell lines and xenografts.

**Methods:** Trop-2 expression was assessed in 90 formalin-fixed-paraffin-embedded tumors and nine primary tumor cell lines by immunohistochemistry (IHC) and flow cytometry, respectively. Trop-2 expression and cell viability after exposure to SG in primary tumor cell lines, non-targeting control ADC, and SG-parental antibody hRS7 were evaluated using flow-cytometry-based-assays. Antibody-dependent-cell-cytotoxicity (ADCC) against Trop-2+ and Trop-2– EOC cell lines was tested *in vitro* using 4 h Chromium-release-assays. *In vivo* activity of SG was evaluated against Trop-2+ EOC xenografts.

**Results:** Moderate-to-strong staining was seen in 47% (42/90) of ovarian tumors by IHC while 89% (8/9) of the primary EOC cell lines overexpressed Trop-2 by flow cytometry. EOC Trop-2+ were significantly more sensitive to SG compared to control ADC (*p* < 0.05). Both SG and hRS7 mediated high ADCC activity against Trop-2+ cell lines. SG also induced significant bystander killing of Trop-2– tumor cells admixed with Trop-2+ EOC cells. In *in vivo* experiments SG treatment demonstrated impressive anti-tumor activity against chemotherapy-resistant EOC xenografts.

**Conclusion:** SG demonstrates remarkable preclinical activity against biologically aggressive and chemotherapy-resistant EOC cell lines and a significant bystander effect against EOC cell lines with heterogenous Trop-2 expression. Clinical trials are warranted.

## Background

Epithelial ovarian cancer (EOC) remains the most lethal gynecologic malignancy. Approximately 14,000 ovarian cancer deaths are estimated in 2019 in the United States alone (1, 2). The use of platinum-based chemotherapy before (i.e., neoadjuvant) or after maximal surgical cytoreduction remains the first line of treatment for advanced stage disease. Unfortunately, most patients eventually develop a chemotherapy-resistant recurrence with an overall 5-year survival rate of 25–30% (3). The development of novel treatment modalities for patients diagnosed with advanced/recurrent EOC are eagerly awaited.

Antibody drug conjugates (ADC) represent a novel and promising therapeutic approach for cancer patients which combine antibody targeting of differentially expressed surface proteins/receptors with a toxic payload to allow selective delivery of chemotherapeutic agents to tumor cells. ADC may optimize tumor targeting *in vivo* while potentially minimizing the side effects of highly toxic chemotherapy agents (4, 5). One of the main challenges in ADC development is the generation of linkers able to maintain the integrity of ADC in human system and allowing the release of the toxic payload after internalization or in close proximity, of the targetcells (5–7).

The TACSTD2 gene on chromosome 1p32, encodes the human trophoblast cell surface antigen 2 (Trop-2), a 46 kDa transmembrane glycoprotein. Many epithelial human tumors differentially express Trop-2 (8). While the functions of this protein in human tumors is not completely understood. Trop-2 overexpression is considered an independent poor prognosis marker in multiple human neoplasia including ovarian cancer by promoting increased proliferation, invasion and metastases (9, 10). Importantly, the differential expression in tumor cells when compared to normal tissues makes Trop-2 be a promising target for cancer immunotherapy.

Sacituzumab govitecan or IMMU-132 (SG) is a novel ADC combining the humanized RS7 antibody targeting Trop-2 coupled to a hydrolyzable linker that allows for a time dependent release of the payload to deliver SN-38, the active metabolite of irinotecan (7-ethyl-10-hydroxycamptothecin) to tumor tissue. SN-38 is significantly more potent than irinotecan (100- to 1,000-fold) and SG antibody is conjugated to the toxic payload with a high ratio (8:1) without affecting targeting and pharmacokinetics (8, 11, 12). The hydrolysable pH-sensitive cleavable linker of SG may not only target Trop-2 when is highly overexpressed in human tumors but also induce a strong bystander effect against Trop-2 negative tumor cells (13). While no report currently exists in the literature on the potential clinical activity of SG in ovarian cancer patients, encouraging therapeutic activity of SG has recently been reported against multiple human tumors including triple negative breast cancer (phase I\II study), urothelial cancer, SCLC and NSCLC (14–17).

The objective of this original research was to evaluate the expression of Trop-2 in EOC tissues and primary cell lines and to examine the preclinical anti-tumor activity of SG *in vitro* and *in vivo* against multiple primary EOC models and xenografts. We demonstrate for the first time that SG is highly active, both *in vitro* as well as *in vivo*, against biologically aggressive EOC and well tolerated in animals bearing EOC chemotherapy-resistant xenografts.

## Methods

### Establishment of High Grade Serous Ovarian Cancer Cell Lines

The study protocol was approved by the Yale Human Investigation Committee and conducted in accordance with recognized ethical guidelines. Informed written consents from cancer patients and healthy donors were obtained following institutional guidelines prior to tissue/blood collection. Fresh tumor biopsy samples, and primary EOC cell lines were established and de-identified as described previously (Table 1) (18–20).

**Table 1:** Epithelial ovarian cancer cell lines with demographics, stage, histologic grade, primary site of tumor, Mean fluorescence intensity (MFI), and score for Trop-2.

**Cell Line**	**Age**	**Race**	**FIGO stage**	**Primary site**	**Histology**	**Δ TROP2 MFI**	**Flow cytometry score**
**Ovarian Cancer Cell Lines, Baseline**						
KR(CH)31	69	W	IV	Ovary	High grade serous	260.5	3 +
OVA1	64	W	IA	Ovary	High grade serous	234.04	3 +
OVA3	53	W	IIIA	Ovary	High grade serous	51.56	2 +
OVA4	64	W	IIIC	Ovary	High grade serous	57.33	2 +
OVA6	57	W	IIIC	Ovary	High grade serous	127.37	3 +
OVA10	51	W	IIC	Ovary	Clear cell	171.89	3 +
OVA11	79	W	IC	Ovary	Clear cell	176.93	3 +
OVA13	42	W	IIIC	Ovary	Clear cell	102.48	3 +
OVA14	58	W	IIIC	Ovary	High grade serous	0.5	0

*MFI 0-20, Flow cytometry score 0; MFI 21-50, Flow cytometry score 1+; MFI 51-100, Flow cytometry score 2+; MFI >100, Flow cytometry score 3+. International Federation of Gynecology and Obstetrics (FIGO) staging and grading*.

### Tissue Microarray

A retrospective, stage I-IV ovarian cancer cohort [EOC *N* = 90 formalin-fixed-paraffin-embedded (FFPE) tumors] represented in tissue microarray (TMA) format was used to evaluate TROP-2 expression by immunohistochemistry (IHC). Briefly, as described in previous studies (7), representative areas of primary high-grade serous carcinomas ovarian cancers, in hematoxylin/eosin–stained preparations and 0.6 mm cores were obtained and arrayed in a recipient block. From different areas of each tumor, four different cores were selected and included in the TMAs, to maximize tumor representation and capture possible marker heterogeneity. For histology processing and staining, 5 μm sections were transferred to glass slides before being stained with purified goat polyclonal antibody against the recombinant human Trop-2 extracellular domain (R&D Systems, Inc., Minneapolis, MN; diluted 1:100) for 1 h. A secondary biotinylated anti-goat antibody (Vector Laboratories, Burlingame, CA; diluted 1:250) and streptavidin–biotin complex (StreptABComplex/HRP, Dako, CA) were then applied, before the use of 303-diaminobenzidine (Dako) as chromogen. Appropriate negative and positive controls were used. The percentage of tumor cells with membranous Trop-2 immunoreactivity was estimated and the staining intensity was assessed semi-quantitatively as follows: 0, no staining; 1+, weak; 2+, moderate and 3+, strong staining. The final immunoreactivity score was calculated by multiplying the staining intensity (1+, 2+, 3+) with the percentage of positive tumor cells, and was classified in four ordinal categories: 0–9 negative (score 0), 10–99 weak (score 1), 100–199 moderate (score 2), and 200–300 strong (score 3).

### Determination of Trop-2 Expression in Primary Ovarian Cancer Cell Lines

Primary EOC cell lines were analyzed by flow cytometry for Trop-2 expression by incubating single cell tumor suspensions with 2.5 μg/mL of primary antibody hRS7 IgG for 120 min at 7 degrees Celsius. A fluorescein isothiocyanate-conjugated goat anti-human F(ab1)2 immunoglobulin (FITC) was used as a secondary antibody (BioSource International, Camarillo, CA). Data were then acquired on Cell Quest software (BD Biosciences, San Diego, CA) with a FACScalibur. Mean fluorescence intensity (MFI) was evaluated using Cell Quest (BD Biosciences) and Prism 8. Cell lines with MFI >100 were determined to have 3+ expression of Trop-2 while cell lines with an MFI of 51–100 were noted to have 2+, 21–50, 1+ and 20 or less was 0.

### Drugs

SG (hRS7-CL2A-SN-38), non-targeting control ADC (h679-CL2A-SN-38), and unconjugated antibody hRS7 IgG were obtained from Immunomedics, Inc. (Morris Plains, NJ). SG and control ADC were dissolved in sterile 0.9% sodium chloride as a 2 μM stock solution for the *in vitro* experiments. Drug-to-antibody ratio (DAR) of SG and control ADC was 6.78 and 6.84, respectively. For the *in vitro* experiments, the dosage of the drug was adjusted according to the DAR, in order to expose equivalent quantities of SN-38 cells treated with SG and control ADC. In *in vivo* experiments, SG and control ADC were dissolved in sterile 0.9% sodium chloride as a 5 mg/mL solution. hRS7 IgG (molecular weight: 150 kDa) was obtained in liquid form from Immunomedics, Inc. as a 10 mg/mL solution.

### Antibody-Dependent Cell-Medicated Cytotoxicity (ADCC)

Ficoll–Hypaque-separated peripheral blood lymphocytes (PBLs) from several healthy donors, were used in standard 4-h chromium (^51^Cr) release assays to measure the cytotoxic reactivity in combination with SG, control ADC, or hRS7 against the EOC cell lines at the effector to target ratio (E:T) of 5:1 and 10:1. The release of ^51^Cr from target cells was measured as evidence of tumor cell lysis after exposure of the tumor cells to a concentration of 2.5 μg/mL of SG, control ADC, or hRS7. Negative controls included tumor cells incubated with PBLs in the absence of ADC. One percentage sodium dodecyl sulfate (SDS) was used to achieve complete lysis of target cells (i.e., 100% lysis), as positive control condition. Chimeric anti-CD20 mAb rituximab (2.5 μg/mL) was also used in all bioassays as additional negative control. ^51^Cr-released from the target cells by cytolysis was counted using a gamma radiation counter (2470 WIZARD2 Automatic Gamma Counter, PerkinElmer). The percentage cytotoxicity of SG, control ADC, or hRS7 was calculated with the formula: % cytotoxicity = 100 × (E–S)/(T–S) (E is the experimental release, S is the spontaneous release by target cells, and T is the maximum release by target cells lysed with 0.1% SDS). Results are presented as mean ± SEM.

### Flow-Cytometry Based Cytotoxicity

Briefly, EOC cell lines were plated in six-well tissue culture plates at a density of 30,000–50,000 cells/well in RPMI 1640 media supplemented with 10% FBS, 1% penicillin/streptomycin, and 1% amphotericin. After 24 h cells were treated with SG, non-targeting control ADC (h679-CL2A-SN-38) and hRS7 at concentrations of 0.2, 0.5, 1, 2, 4 nM (adjusted according to DAR) for 12 h before being washed with culture medium to remove any unbound ADC or naked Mab. The 6-well plates were then incubated for 72 h (i.e., an incubation time demonstrating optimal SG efficacy combined with low control antibody background cytotoxicity) before being harvested and incubated with propidium iodide (2 μl of 500 μg/mL stock solution in PBS). The viable cells were then quantified using a previously characterized flow-cytometry based assay (21). In some flow cytometry experiments, tumor cells were also stained with Annexin V-fluorescein isothiocyanate (Annexin V-FITC) and propidium iodide (PI) double staining to quantify apoptosis vs. necrosis, respectively. To determine the IC_50_ of SG, control ADC, and hRS7, every experiment was repeated a minimum of three times per cell line.

### Bystander Effect

Briefly, a Trop-2 negative uterine serous cell line (i.e., ARK4) stably transfected with a Green Fluorescence Protein (GFP) plasmid (pCDH-CMV-MCSEF1-copGFP, a gift from Dr. Simona Colla, MDACC) (7), was plated alone (80,000 cells/well) or mixed with KRCH31 (3+ Trop-2 expressing EOC cell line) (40,000 ARK4 cells/well and 20,000 KRCH31) in 6-well plates (2 mL/well). After overnight incubation, cells were treated with 1 nM of SG or control ADC (h679-CL2A-SN-38) for 12 h, as described above. After 72-h, cells were harvested, incubated with propidium iodide (2 μl of 500 μg/mL stock solution in PBS) and counted by flow cytometer, to evaluate ARK4 cells viability (mean ± SEM) in each well (i.e., percentage of live vs. dead cells present in each well). Final assessment of the bystander effect was performed by comparing the percentage of live Trop-2 negative cells (GFP-ARK4) when they were co-cultured with Trop-2 positive cells (KRCH31) and treated with either SG vs. the control ADC.

### *In vivo* Testing

KRCH31, a previously characterized (19, 22) high grade serous ovarian cancer cell line, derived from a patient harboring chemotherapy-resistant disease overexpressing Trop2+, was established as a xenograft model into 5–8-week-old severe combined immunodeficiency (SCID) mice (ENVIGO, Indianapolis, IN). Animals were subcutaneously injected with 5 million KRCH31 cells, suspended in 300 μL of PBS containing cells. Matrigel® (BD Biosciences) was admixed to tumor cells to improve cells growth in mice. When the tumor volume reached 0.3 cm^3^, mice were randomized into four groups (six mice/group): saline control, SG, control ADC, and hRS7. SG, control ADC, and hRS7 were administered at the dose of 500 μg IV twice per week for 3 weeks. Tumors were measured twice weekly. Tumor volume was determined using the formula (A^2^ × B)/2, where B represented the largest tumor diameter size and A was the smaller perpendicular tumor diameter. Mice were humanely euthanized when the tumor volume reached 1.5 cm^3^ or when they reached the end of 60 days of follow up. The Institutional Animal Care and Use Committee (IACUC) rules were used for animal care and euthanasia in all experiments.

### Statistical Analysis

Statistical analysis was performed using Graph Pad Prism 8 (GraphPad Software, Inc. San Diego, CA). The differences in the inhibition of proliferation in the EOC cell lines after exposure to treatments were evaluated by two-tailed unpaired student *t*-test. Tumor volume differences at specific time points were compared using an unpaired *t*-test. Overall survival data were analyzed using the Kaplan-Meier method. Survival curves were compared using the log-rank test. A two-sided *p* < 0.05 was considered to be significant (7).

## Results

### Trop-2 Expression in Ovarian Cancer Patient Samples by TMA

**Figure 1:**
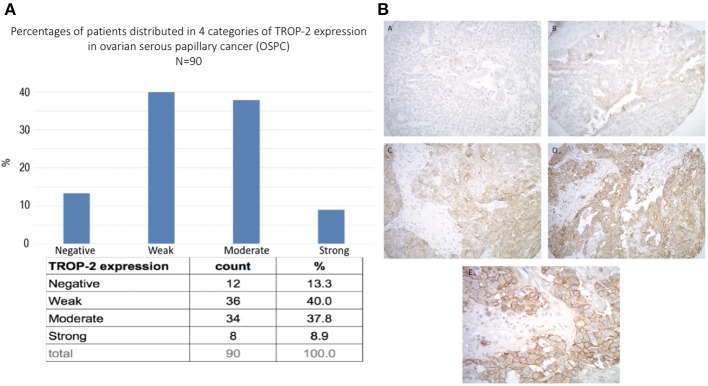
**(A)** Trop-2 expression in tissue micro array of 90 EOC samples. **(B)** Representative IHC images from the tissue microarray show no Trop-2 immunostaining (score 0) (A), weak focal (score 1) (B), moderate focal (score 2) (C) and strong diffuse (score 3) (D) membranous Trop2 expression. (E) higher magnification image shows membranous localization of Trop2. Original magnification: 200x (A–D) and 400x (E).

A tissue microarray containing 90 ovarian high-grade serous carcinoma samples was used to semi-quantitatively analyze Trop-2 expression by IHC. Moderate-to-strong (scores 2+/3+) Trop-2 expression was seen in 47% (42/90) of the tumors (Figure 1A). Representative IHC images of the immunohistochemical score range are presented in Figure 1BA–D. Please note in the higher magnification image (Figure 1E) the membranous localization of Trop2 in ovarian cancer cells.

### Trop-2 Expression in Primary Ovarian Cancer Cell Lines by Flow Cytometry

**Figure 2:**
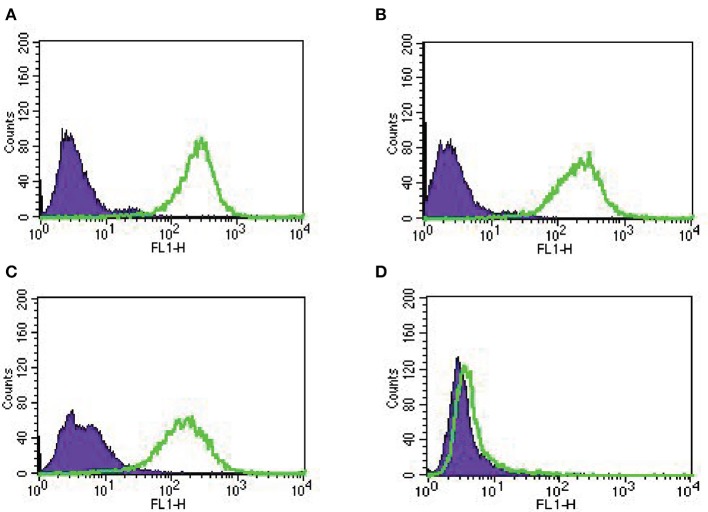
Representative Trop-2 expression by flow cytometry histograms of primary EOC cell lines, **(A)** KRCH31, **(B)** OVA1 and **(C)** OVA10 3+ Trop-2 expression, **(D)** OVA14 0 Trop-2 expression.

In our study, we established nine primary EOC cell lines from fresh tumor biopsy samples as described in Methods. Stage, grade, histology and primary site of the tumors are displayed in Table 1. Eighty-nine percent of the EOC cell lines were determined to have more than 2+ Trop-2 expression by flow cytometry (Table 1). Figure 2 shows four representative flow cytometry histograms of primary EOC cell lines used in the experiments, showing 3+ (A, KRCH31; B, OVA1; C, OVA10 cell lines) and 0 (D, OVA14 cell line) Trop-2 expression.

### SG and hRS7 IgG Induce Strong ADCC Against Trop2-Positive Primary EOC

**Figure 3:**
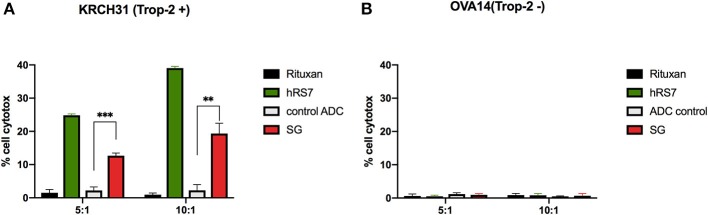
ADCC (mean ± SEM) of SG, control ADC isotype, hRS7 IgG and Rituximab (anti-CD20) in two representative EOC cell lines [i.e., **(A)** KRCH31 3+ Trop-2 positive cell line vs. **(B)** OVA14 Trop-2 negative cell line]. Significant ADCC was detectable only against the Trop-2 positive tumors (*p* < 0.05, *P ≤ 0.05, **P ≤ 0.01, ***P ≤ 0.001).

As described in methods, we tested two selected primary EOC cell lines (i.e., KRCH31 Trop-2 + and OVA14 Trop-2–) for their sensitivity to PBL-mediated cytotoxicity in standard 4-h ^51^Cr release assays. Both ovarian cancer cell lines were highly resistant to ADCC when treated with the isotype control antibody (Rituxan) (2 μg/mL) at E:T ratios of 5:1 and 10:1 (i.e., mean ± SEM, cytotoxicity of 1.2 ± 0.3% for KRCH31 and 0.7 ± 0.15% for OVA14 in the presence of Rituxan + PBL) (Figure 3). Subsequently, we studied ADCC on EOC cell lines by combining heterologous PBLs with SG, control ADC, and naked antibody hRS7-IgG at 2 μg/mL. hRS7 and SG but not control ADC demonstrated marked ADCC against Trop-2 positive primary EOC cell lines (i.e., KRCH31). As depicted in Figure 3A, a mean cytotoxicity ± SEM = 16.01 ± 3.3% for SG and 31.9 ± 7.1% for hRS7, was demonstrated, respectively, while low/negligible killing (i.e., 2.2 ± 0.01%) was induced by control ADC (*p* < 0.001 at 5:1 ratio, Figure 3A). In contrast, neither hRS7 nor SG and control ADC, induced significant ADCC against the TROP-2 negative cell line OVA14 (Figure 3B).

### *In vitro* Viability Assays With SG, Non-targeting Control ADC and hRS7 IgG in Primary EOC Cell Lines

**Figure 4:**
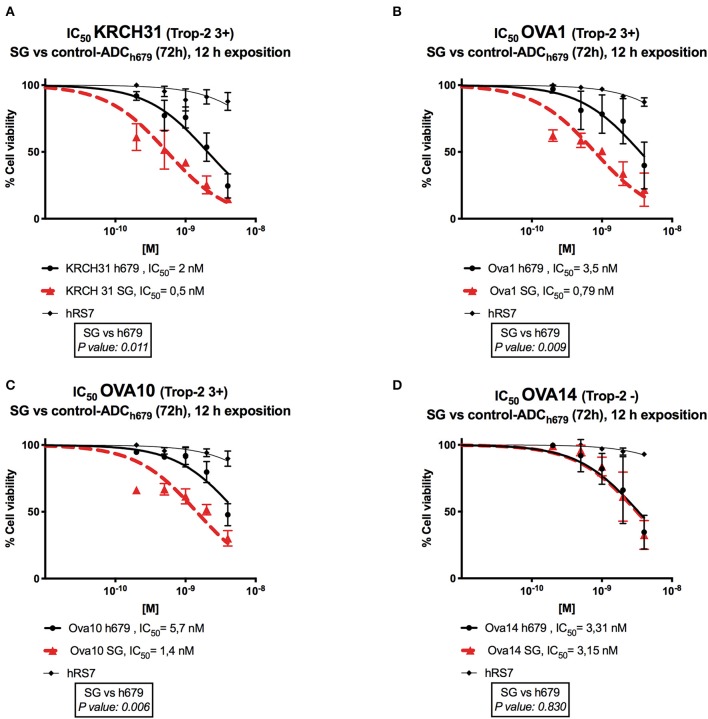
Determination by IC_50_ of SG cytotoxicity compared to controls, control ADC (h679-CL2A-SN-38) and hRS7 IgG in EOC. **(A–C)** EOC cell lines with high Trop-2 expression (3+) (i.e., KRCH31, OVA1, OVA10) demonstrated significantly lower IC_50_ when compared to control ADC *(p* < 0.05*)*. **(D)** EOC cell line with low/negligible Trop-2 expression (i.e., OVA14) showed no difference in the IC_50_s of SG and control ADC. hRS7 antibody was inactive against all these cell lines.

**Figure 5:**
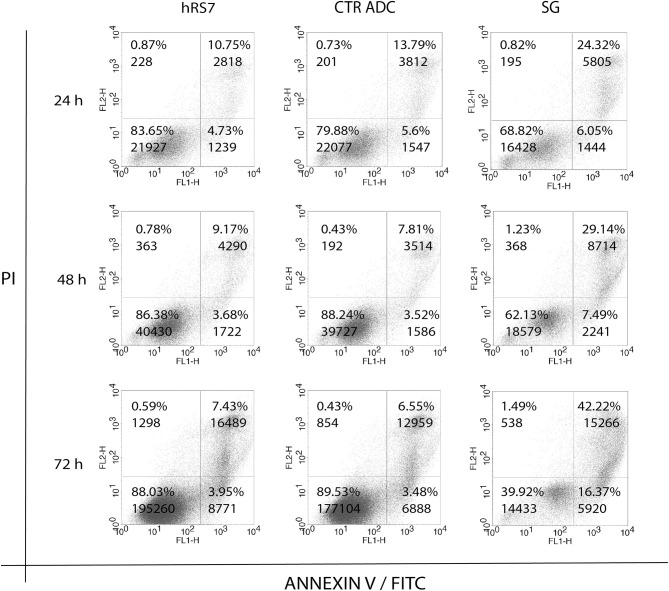
Time dependent cytotoxicity of hRS7, control ADC and SG after 24, 48, and 72 h exposure. Down right and up left quadrants show single positive events for FL1-H (ANNEXIN V-FITC, apoptosis) and FL2-H (Propidium Iodide, necrosis), respectively. Up right quadrant shows double positive events for FL1-H and FL2-H, respectively. Double positive events stand for tardive apoptosis. Maximal cytotoxicity of SG was demonstrated after 72 h exposure.

Next, we exposed four representative primary EOC cell lines (i.e., 3 Trop-2 + and one 1 Trop-2 –) to scalar concentrations of SG, non-targeting control ADC and hRS7 IgG. The IC_50_ of SG, control ADC, and hRS7 for each EOC cell lines was determined. As shown in Figure 4, SG was significantly more potent against Trop-2 positive EOC cell lines (i.e., a 4.0-, 4.07-, and a 4.43-fold increase in cell cytotoxicity against KRCH31, OVA10 and OVA1, respectively) when compared to the control ADC (*p* < 0.05) (Figures 4A–C). In contrast, when a Trop-2 negative cell line (i.e., OVA14) was challenged with SG, no increase in cell cytotoxicity was detected when compared to the control ADC (*p* = 0.830) (Figure 4D). The IC_50_ of hRS7 (i.e., naked Ab) was more than 20-fold higher than SG IC_50_ for all the cell lines tested. To gain better insight into the mechanism of SG activity against ovarian cancer cells, in some experiments KRCH31 tumor cells were exposed to the ADC and control antibody for 24, 48, and 72 h before being harvested for Annexin V and PI staining. As representatively shown in Figure 5, maximal SG induced apoptosis in the presence of low background control antibody cytotoxicity (i.e., hRS7 IgG and control ADC) was detected after 72 h exposure.

### Bystander Effect *in vitro*

**Figure 6:**
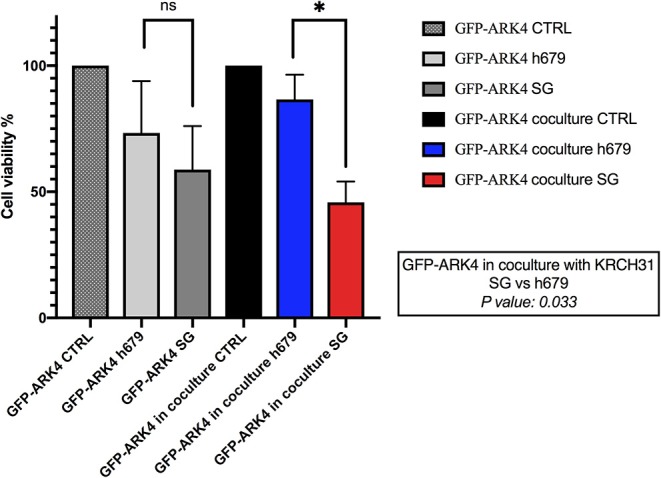
Bystander assay. Black bar: Low/negative Trop-2 expressing ARK4 cells (GFP-ARK4 cells), co-cultured without any treatment (control, CTRL). Blue bar: low Trop-2 expressing cells (GFP-ARK4 cells) co-cultured with high Trop-2 expressing cells (KRCH31), and treated with control ADC at 1 nM concentration. Red bar: low Trop-2 expressing cells (GFP-ARK4 cells) co-cultured in with high Trop-2 expressing cells (KRCH31), and treated with SG at 1 nM concentration. Significant increase in ARK4 cell cytotoxicity was detected with SG treatment only at the time of the co-incubation with KRCH31 cells (*p* < 0.05, *P ≤ 0.05, **P ≤ 0.01, ***P ≤ 0.001).

The ability of SG to induce a bystander killing effect against EOC with heterogeneous Trop-2 expression was tested by admixing KRCH31 EOC cells (i.e., high Trop-2+ expression) *in vitro* with the low/negligible Trop-2 expressing cell line (i.e., GFP-ARK4). As shown in Figure 6, a significant increase in bystander cytotoxicity of Trop-2- cells was seen only when GFP-ARK4 and KRCH31 were treated as co-cultures with 1 nM of SG comparing to control ADC treatment (*p* < 0.05). No difference was seen in GFP-ARK4 cytotoxicity in monocultures.

### *In vivo* Antitumor Activity

**Figure 7:**
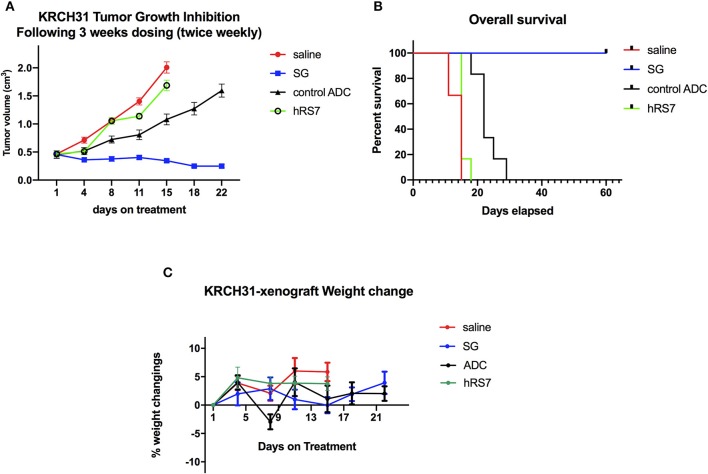
*In vivo* efficacy of SG: **(A)** Antitumor activity of SG was compared to controls including, control ADC isotype, hRS7 IgG (naked Ab) and saline (Control), in EOC xenograft models (i.e., KRCH31, 3+ Trop-2 positive). Mice were treated intravenously with twice weekly doses for 3 weeks as described in Methods. A significant difference in tumor growth inhibition was detected beginning on day 11 (*p* < 0.001) in SG-treated group when compared to the other control groups. **(B)** Overall survival. Median survival for SG group was 60 days, compared to 22 days for control ADC and 15 days for hRS7 and Control. **(C)** Mice weight change during treatment.

*In vivo* experiments comparing the antitumor activity of SG, control ADC, hRS7 IgG and normal saline (Control) were conducted using the Trop-2+ chemotherapy-resistant EOC xenograft (i.e., KRCH31). Treatment with SG showed a statistically significant difference in tumor growth inhibition when compared to control ADC, saline and hRS7 starting at day 11 of the treatment (*p* < 0.05). The *P*-value after day 11 of the treatment remained highly significant (i.e., *p* < 0.0001) until all mice treated with control ADC, saline and hRS7 were euthanized (Figure 7A). Median survival time (MST) for the SG group was >60 days with all of the treated mice still alive at the time the study was ended on day 60 (Figure 7B), compared to no long-term survivors in any of the control groups (MST = 22 days for control ADC and 15 days for the hRS7 and saline controls (Figure 7B) (*p* < 0.05). The tolerability of SG treatment was monitored and no significant change in mice body weight was detected at any time point during the experiments (Figure 7C). Consistent with these results, necropsy and tissue evaluation did not demonstrate any significant sign of toxicity in animals after SG exposure (data not shown).

## Discussion

Almost 40 years ago, the Trop-2 glycoprotein was first described as a cell surface marker of trophoblast cells (23). Since then, Trop-2 has been characterized as a transmembrane-calcium-signal-transducer differentially expressed in multiple human epithelial tumors and conferring tumor cells increased proliferation and cell migration (24–27). Trop-2 overexpression has been also demonstrated to affect intracellular Bax/Bcl-2 balance, yielding a powerful anti-apoptotic effect (28). Since Trop-2 is differentially expressed on the surface of a variety of human epithelial tumors when compared to normal cells, it may represent a promising target for targeted therapeutics such as ADCs.

Accordingly, in this study we evaluated Trop-2 expression level in 90 high-grade serous ovarian carcinomas and tested for the first time the activity of SG, against nine primary ovarian cancer cell lines, with serous and clear cells histology, with differential Trop-2 expression. We report moderate-to-strong Trop-2 expression in 47% (42/90) of EOC samples evaluated by IHC, and 89% (8/9) of the primary EOC cell lines evaluated by flow cytometry. Importantly, we demonstrate high *in vitro* sensitivity to SG exposure in all Trop-2 positive EOC cell lines tested when compared to control ADC and naked antibody. In contrast, the Trop-2 negative EOC cell line (OVA14) was not significantly affected by SG when compared to control ADC isotype. These results demonstrate the need for Trop-2 protein/receptor expression on tumor cells for the induction of SG cytotoxic activity against EOC.

In our experiments, we also demonstrated that tumor cell killing is also potentially mediated by immune system cells (i.e., NK cells). Indeed, both SG and hRS7-IgG demonstrated significant ADCC against Trop-2 positive EOC cell lines in the presence of PBL while non-targeting control ADC generated only low levels of cytotoxicity. The killing activity was Trop-2 specific as demonstrated by the negligible ADCC induced by SG against a Trop-2 negative tumor cell line (OVA14). These findings confirm and extend previous results from our group evaluating the activity of the unconjugated humanized anti-Trop-2 monoclonal antibody hRS7 (29).

Ovarian cancer is a heterogeneous disease and accordingly, expression of Trop-2, similarly to other surface markers (30), may not be uniformly expressed in EOC. Importantly, SN-38 may be delivered in tumors intracellularly as well as in adjacent tumor cells not directly targeted by the ADC through its hydrolysable SG linker. We consistently found SG to induce a significant bystander killing against Trop-2 negative tumor cells only when admixed with Trop-2 overexpressing tumor cells. These results strongly suggest that SG may also be active against recurrent EOC patients harboring heterogeneous Trop-2 expression.

Finally, in *in vivo* experiments with Trop-2 + EOC xenografts established from a patient with a chemotherapy-resistant disease, we demonstrate that injections of SG twice-weekly for 3 weeks induced a statistically significant difference in KRCH31 tumor growth inhibition when compared to control ADC. Of interest, SG treatment *in vivo* was well tolerated, with no significant weight change documented in animals for the entire study. Taken together these results demonstrate for the first time, *in vivo* activity of SG against biologically aggressive and chemotherapy-resistant EOC and strongly support the use of this ADC in future clinical trials.

While the efficacy of SG in ovarian cancer patients is currently unknown, preliminary SG clinical activity has recently been reported against multiple human tumors (15, 16, 31). For example, Bardia et al., in a phase 1/2 single-group, multicenter trial, demonstrated that SG treatment provided durable objective responses in 1/3 of pretreated metastatic triple-negative breast cancer patients enrolled in the study (17). Importantly, our group has recently reported a remarkable clinical response of SG treatment in a 74-year-old woman with recurrent and widespread treatment-resistant uterine serous carcinoma (66% reduction of target lesions by RECIST 1.1 with a duration response of over 10 months) (32). SG was well tolerated with limited adverse treatment-related events (14, 15, 17, 31). Grade ≥ 3 neutropenia was the most common side effect (30–42%) reported in clinical trials, but neutropenic fever occurred only in 2–9% of patients (15–17). As result of these findings, SG has received Breakthrough Therapy Designation from the U.S. FDA for the treatment of TNBC patients who have failed at least 2 prior therapies for metastatic disease.

In conclusion, our results demonstrate that (1) Trop-2 is overexpressed in ~50% of EOC, (2) primary EOC cell lines overexpressing Trop-2 are sensitive to killing *in vitro* by SG, (3) SG in the presence of effector cells (NK cells) may induce significant ADCC against Trop-2 positive EOC cells, (4) SG has a significant bystander killing effect, which could aid in the treatment of tumors with heterogeneous antigen expression, and (5) chemotherapy-resistant EOC xenografts overexpressing Trop-2 are highly sensitive *in vivo* to SG. These preclinical results combined with recent phase I/II IMMU-132-01 basket study data demonstrating encouraging clinical activity in multiple human solid tumors resistant to chemotherapy, strongly support the design of clinical trials in Trop-2 positive recurrent EOC patients.

## Precis

The antibody-drug-conjugate sacituzumab govitecan demonstrated significant preclinical activity against primary Trop-2+ EOC cell lines and xenografts.

## Data Availability Statement

All datasets for this study are included in the article/supplementary material.

## Ethics Statement

The animal study was reviewed and approved by Institutional Animal Care and Use Committee (IACUC).

## Author Contributions

AS designed research. EP, SL, SP, BZ, SB, AB, LZ, AM, EB, PM, NB, GA, CH, GM, ER, D-AS, JT-R, PH, and MA performed research. PS and GS contributed new reagents and analytic tools. AS and EP wrote the paper.

### Conflict of Interest

AS declares research funding support to Institution from Immunomedics, Inc. AM declares that she receives research funding support from Spanish Medical Oncology Society. The remaining authors declare that the research was conducted in the absence of any commercial or financial relationships that could be construed as a potential conflict of interest.
